# Pre-crop Values From Satellite Images for Various Previous and Subsequent Crop Combinations

**DOI:** 10.3389/fpls.2019.00462

**Published:** 2019-04-09

**Authors:** Pirjo Peltonen-Sainio, Lauri Jauhiainen, Eija Honkavaara, Samantha Wittke, Mika Karjalainen, Eetu Puttonen

**Affiliations:** ^1^Natural Resources Institute Finland (Luke), Helsinki, Finland; ^2^Natural Resources Institute Finland (Luke), Jokioinen, Finland; ^3^Finnish Geospatial Research Institute (FGI), National Land Survey of Finland (NLS), Masala, Finland; ^4^Department of Built Environment, Aalto University, Espoo, Finland

**Keywords:** crop rotation, crop sequencing, NDVI, pre-crop effect, remote sensing, Sentinel-2

## Abstract

Monocultural land use challenges sustainability of agriculture. Pre-crop value indicates the benefits of a previous crop for a subsequent crop in crop sequencing and facilitates diversification of agricultural systems. Traditional field experiments are resource intensive and evaluate pre-crop values only for a limited number of previous and subsequent crops. We developed a dynamic method based on Sentinel-2 derived Normalized Difference Vegetation Index (NDVI) values to estimate pre-crop values on a field parcel scale. The NDVI-values were compared to the region specific 90th percentile of each crop and year and thereby, an NDVI-gap was determined. The NDVI-gaps for each subsequent crop in the case of monocultural crop sequencing were compared to that for other previous crops in rotation and thereby, pre-crop values for a high number of previous and subsequent crop combinations were estimated. The pre-crop values ranged from +16% to -16%. Especially grain legumes and rapeseed were valuable as pre-crops, which is well in line with results from field experiments. Such data on pre-crop values can be updated and expanded every year. For the first time, a high number of previous and following crop combinations, originating from farmer’s fields, is available to support diversification of currently monocultural crop sequencing patterns in agriculture.

## Introduction

Monotonous crop sequencing and simplified crop rotations imply that many ecosystem services and potential environmental benefits – both attainable by more diverse land use – remain unutilized. Though highly dependent on crops, their sequencing patterns and growing conditions (Sieling and Christen, 2015), frequently reported benefits of diversified crop rotations include reduced nitrogen (N) application rates (Jensen et al., 2010; St. Luce et al., 2015; Xing et al., 2017; Weiser et al., 2018), as well as improved soil structure, soil carbon, pH and soil functionality, e.g., through changes in soil microbial communities (Robson et al., 2002; Persson et al., 2008; Wang et al., 2012; Aschi et al., 2017; Prade et al., 2017). Furthermore the benefits also include suppressed disease, pest and weed infestation and thereby less intensive pesticide use (Bankina et al., 2013; Andert et al., 2016), in addition to providing higher yields of subsequent crops (Weiser et al., 2018), though not necessarily improved yield stability (St-Martin et al., 2017). According to a retrospective analysis, the major productivity shifts in dry-land cereal production regions of Australia coincided with changes in crop management including crop sequencing patterns (Kirkegaard and Hunt, 2010).

Despite numerous benefits from diversification, lack of sufficient encouragement and incentives in the prevailing market conditions hinder the abandonment of cereal dominated arable systems (Preissel et al., 2015; Zander et al., 2016) as does the insufficient capability to value the benefits of diversified crop sequencing in order to reassure farmers. Pre-crop value is a measure to identify how beneficial different previous crops are for a subsequent crop in rotation. This is often expressed as higher yield or biomass compared to monocultural crop sequencing (positive pre-crop value). However, yield loss is also possible in the case that the previous crop and the following crop are incompatible in a way or another (negative pre-crop value). Variation in the pre-crop value is dependent on growing conditions. For example, variation in weather events impacts crop growth and soil processes that contribute to the pre-crop value (Sieling and Christen, 2015). The availability of N for the crop preceded by legumes or rapeseed and thereby, the capacity to replace N fertilizers with residual N (Kirkegaard et al., 1997; St. Luce et al., 2016; Weiser et al., 2018) calls for strategies to safeguard the environment from possible additional N loads in subsequent years (Jensen et al., 2010; Grant et al., 2016; Plaza-Bonilla et al., 2017). Also, the long-term impacts of diverse crop sequencing patterns should be acknowledged (Robson et al., 2002), although these may be harder to quantify as such data is available only from long-term experiments.

Shifting from monocultural crop sequencing to more diverse crop rotations including grain legumes provides both environmental and economic benefits (Lötjönen and Ollikainen, 2017). The pre-crop value is what the farmer perceives as an economically important benefit. Crop sequencing has instant impacts on the farming economy (Xing et al., 2017). However, economic optimization models with crop rotations even as the core management practice in a farm (Liu et al., 2016) often lack precise information on the pre-crop value, its variability and drivers, and therefore, cautious estimations may underestimate the benefits of break-crops in rotations.

The determination of the pre-crop value should be based on long-term experiments (Sieling and Christen, 2015). The applicability of the data beyond the study region can be enhanced by meta-analyses based on a high number of published experiments so that the merged data characterizes both spatial and temporal variation in pre-crop values (Angus et al., 2015; Preissel et al., 2015). However, usually the value is determined for one, sometimes two subsequent cropping years in the rotation (Tamm et al., 2016). Such meta-analyses are, however, often forced to concentrate on only one subsequent crop, which is typically wheat (*Triticum aestivum* L.) (Angus et al., 2015). Hence, despite a long history of crop sequencing and a substantial set of experiments and data evidencing the value of crop sequencing compared to monocultural rotations (Angus et al., 2015), there is a knowledge gap in understanding the benefits of diversification for a high number of subsequent crops (McEven et al., 1989; Jensen et al., 2010). This gap needs to be closed to thoroughly support farmers in their decision making on land use.

**FIGURE 1:**
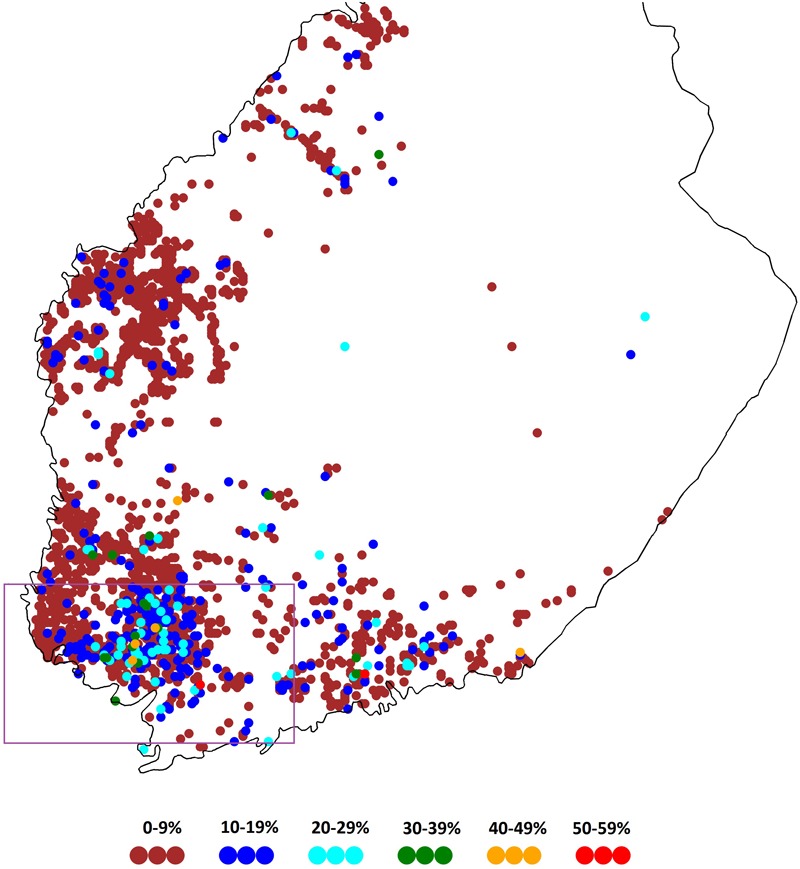
The framed study region with the highest number of crop choices for cultivation in Finland. Each dot indicates the share of the diverse crop rotations in the 2 × 2 km area in the case that the area has ≥30 field parcels.

Remote sensing data provides largely unexploited opportunities in agriculture to assess land use and crop growth on parcel, farm and regional scales. This is especially relevant for cases where systematic data is lacking or scattered. This sort of data has been applied for the identification of crop species, crop sequencing patterns and soil management practices (Peña-Barragán et al., 2011; Conrad et al., 2016; Mueller-Warrant et al., 2016; Waldhoff et al., 2017). With this study we aimed to take a step further and provide a large-spectrum of pre-crop values for various pre-crop and subsequent crop combinations by using *Sentinel-2* satellite images and the derived mean values from the Normalized Difference Vegetation Index (NDVI). The NDVI-data was combined with existing data on crops grown in each field parcel for a total of 2,173,296 field parcels of the prime crop production region of Finland (Figure 1). Thereby, our aim is to support diversification especially as cereal rotations dominate agricultural land use in Finland (Peltonen-Sainio et al., 2017, 2018). Furthermore, the main drivers for temporal and spatial variation in pre-crop values were characterized by supplementing data on pre-crop benefits with data on weather, soil, crop rotation, farm type, farm size and field parcel characteristics. Following these principles our ultimate aim was to provide a set of condition dependent, dynamic pre-crop values. The added value is expected when all the data originates from farmers’ fields: this better facilitates implementation compared to the data achieved with field experiments, because the latter might overestimate the pre-crop benefits when compared to the “true farm environment” (Weiser et al., 2018). Nonetheless, such experiments are non-existent for Finland.

## Materials and Methods

### Crop Choices and Their Allocation to Field Parcels

We used data from the Agency of Finnish Food Authority^1^ on the allocation of field parcels for different crops in the prime crop production region of Finland (Figure 1) to identify all possible previous crop and subsequent crop combinations in the study region. The aim was to assess the pre-crop values. For this purpose, crop species data was linked to the NDVI-values derived from satellite images. Some field parcels had two or more agricultural parcels with different crops. Because the position of the agricultural parcel within the field parcel was unknown, a field parcel was included if the largest agricultural parcel covered at least 70% of the area of the field parcel. Typically, a field parcel with more than two agricultural parcels contains a buffer strip at the margin of the field. Thereby, such combined data comprised a total of 120,174 field parcels in 2016 and 118,116 in 2017.

The following crop species or alternative land uses were identified and their value as pre-crops were analyzed: spring barley (*Hordeum vulgare* L.), oats (*Avena sativa* L.) and wheat (*Triticum aestivum* L.), winter wheat and rye (*Secale cereal* L.), spring turnip rape (*Brassica rapa* L.) and oilseed rape (*Brassica napus* L.) (together called as rapeseed hereon), peas (*Pisum sativum* L.), faba beans (*Vicia faba* L.), sugar beet (*Beta vulgaris var. altissima* L.), potatoes (*Solanum tuberosum* L.), caraway (*Carum carvi* L.), linseed flax (*Linum usitatissimum* L.), oilseed radishes (*Raphanus sativus var. oleiformis* L.), bare fallow, stubble fallow, green fallow, land used for green manuring crop, nature managed fields, diverse game fields, perennial grasslands for animal feed, perennial pastures, perennial grasslands for seed production, annual grasslands for animal feed, annual pastures and green forage crops.

The value of different pre-crops was estimated for the following subsequent crops or groups of crops: spring barley, oats, and wheat, winter wheat and rye, rapeseed, peas, faba beans, sugar beet, potatoes as well as perennial and annual production grasslands.

### Satellite Imagery and Derived NDVI-Values

The NDVI values were derived from all available *Sentinel-2* imagery with less than 99% cloud cover from April to October for the years 2016 and 2017. *Sentinel-2* is one of six missions of the Copernicus program, providing multispectral imagery at 13 wavelength intervals (bands) and with a revisit time of three to six days depending on the geographic location. It provides red (665 nm), green (560 nm), blue (490 nm) and near infrared (NIR, 842 nm) bands at a 10 meter resolution, four red-edge (705, 740, 783 and 865 nm) and two short-wave infrared (SWIR, 1610 and 2190 nm) bands at a 20 meter resolution, and three bands for cirrus detection at a 60 meter resolution (Drusch et al., 2012).

**FIGURE 2:**
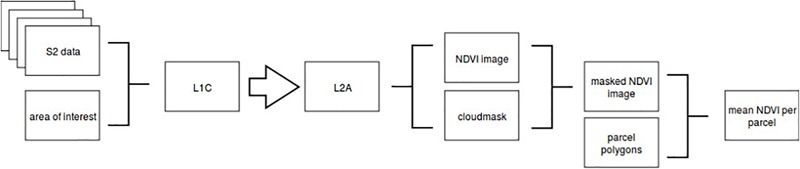
Simplified flowchart of processing pipeline in Finnish Geospatial Research Institute for *Sentinel-2* images.

The study area in South-Western Finland is covered by 4 tiles (34VEN, 34VEM, 34VFN, 34VFM) of the *Sentinel-2* tile system. Each tile has a size of 110 × 110 km with a 10 km overlap. The processing of the 587 scenes was done using a process developed by the Finnish Geospatial Research Institute (FGI) on the EODC platform (“Earth observation data center” (EODC) GmbH, Vienna, Austria) where all *Sentinel-2* data was provided for the L1C “top of atmosphere” level and on-site processing was possible. The L1C products were transformed to “bottom of atmosphere” L2A and cloud mask products using ESA’s *sen2cor* processor^2^. Next, cloud masks and NDVI images were calculated using ESA’s Sentinel Application Platform (SNAP) python interface *snappy*^3^. The NDVI values were calculated as

$$NDVI=\frac{NIR_{842}-Red_{665}}{NIR_{842}+Red_{665}}$$

where both bands captured data at a 10-meter resolution. After masking the NDVI image with the cloud mask, mean NDVI values were extracted per field parcel (given as polygons in the ESRI shapefile format). See also Figure 2 for a simplified flowchart of the process.

### Estimation of Pre-crop Values for Different Pre-crop and Subsequent Crop Combinations

Weather conditions vary significantly within study-area and an NDVI-value of a field is feasible to compare only for fields in close vicinity. An NDVI-value depends on the crop. Therefore, the study area was divided into four sub-areas and an NDVI-value of a crop in a field parcel was compared to a distribution of the NDVI-values of the same crop in field parcels with the same sub-area. These comparisons were made on three pre-selected dates between 1st July and 10th August. The dates were selected separately for each sub-area to minimize the cloud cover of the study area.

For grasslands, three dates were selected between 10th May and 10th June. In Finland, the 1st cut is typically done between 15th and 25th June and the NDVI-values for grass are mutually comparable only before that. Only the NDVI-values before the 1st cut were included because after this the timing of the cutting varies greatly depending on the overall targeted yields and the number of cuttings on a farm, and other variables such as weather conditions and the age of the grassland.

The NDVI-value of a field was converted to an NDVI-gap as follows:

$$gap_{i}=\{\begin{array}{c} 0,\;\;\;\;\;\;\;\;\;\;\;\\ a+bx_{i},\\ c+dx_{i},\; \end{array}\;\begin{array}{c} \;if\;x_{i}\geq g_{90}\;\;\;\;\;\;\;\;\;\;\;\;\\ if\;g_{90}<x_{i}\leq g_{50\;}\\ \;\;if\;x_{i}<g_{50}\;\;\;\;\;\;\;\;\;\;\;\;\;\; \end{array}$$

Where x_i_ is the NDVI-value for the ith field parcel, g_90_, g_50_ and g_25_ are 90th, 50th, and 25th percentiles of the NDVI-distribution for the crop cultivated on the ith field parcel. Parameters a and b are regression coefficients so that the gap is 0 and 0.30 at g_90_ and g_50_, respectively. In the same way, c and d are regression coefficients so that the gap is 0.30 and 0.55 at g_50_ and g_25_, respectively. This means that an NDVI-value higher than the 90th percentile had no NDVI-gap. If an NDVI-value was smaller than the 90th percentile, the gap increases linearly until the NDVI reaches the median (g_50_) where the gap is 0.30. If an NDVI-value was smaller than the median, the gap increases at a slope resulting in a gap of 0.55 then an NDVI-value reached the lowest quarter. These gap-values were obtained from the official yield statistics produced by Luke. Compared to the yield level at the 90th percentile, the yield loss was 30 and 55% for farms located in the median and lowest quarter of the yield level distribution. The definition used for the gap was needed because the variation between field parcels was lower for small and high NDVI-values than values near 0.50. Without this definition, some crops or geographic areas would have had higher gaps than others without any true rationale.

To estimate the pre-crop values of a certain pre-crop and subsequent crop combination, the means of the NDVI-gaps for all the measured gaps in the combination were calculated. The results on pre-crop and subsequent crop combinations were only included if the values occurred at least 20 times in a year. Thereafter the average NDVI-gap for each previous crop and subsequent crop combination was compared to the NDVI-gap in the case that the previous crop and subsequent crop were the same (i.e., monocultural crop sequencing).

The developed NDVI-value based method allows pre-crop value estimation of permanent grassland for other crops, but the method had clear limitations estimating pre-crop values of other crops than perennial grassland because the estimation of the pre-crop value was biased. All the other crops seemed to have negative values compared to production grassland as its own pre-crop (Supplementary Figure S2). This was attributable to the fact that when the other crops were pre-crops, it was the 1st year of the grassland, and after that yields become higher until the grassland is three or four years old. Hence, this developed method can only be applied for the estimation of the pre-crop value of grasslands for other crops.

### Variation in Pre-crop Value and Its Major Drivers

The contribution of the farm, farm size, farm type, farming system (conventional/organic), field parcel characteristics and the pre-crop toward the variation in crop specific NDVI-gaps was estimated. The following field parcel characteristics were considered: field size, field shape [uniformity Peltonen-Sainio et al., 2017], slope, distance of a field parcel to the farm center, the proximity to a waterway, soil type and whether the land was leased or owned by the farmer. From these, the field size, slope and soil type all caused variation in the NDVI-gaps as did the year, soil type × year and pre-crop × year.

This was modeled using a variance component model with the SAS/MIXED software package. The model estimates the variance for each variable and for selected interactions. All variables must be categorical. The variables having impacts were categorized as shown in Table 1. The model was fitted separately for all crops. The coefficient of determination (*R*-square) was calculated by comparing the sum of variances to the total variance. The importance of individual variables and interactions was calculated by comparing the variance of a variable to the sum of variances.

**Table 1:** Sources of identified variation in NDVI-gaps for different arable crops depending on farm and field parcel characteristics, pre-crop and year.

Source	Spring barley	Spring oats	Spring wheat	Winter wheat	Winter rye	Rapeseed	Faba beans	Sugar beet	Potatoes
Farmer	73.3	73.2	66.3	77.2	61.5	72.9	62.2	76.0	67.0
Farm size^a^	1.7	0.5	1.2	2.6	0.0	0.0	1.9	0.0	0.0
Farm type	0.0	3.7	3.9	0.6	12.2	2.1	8.8	0.0	0.0
Conventional/organic	1.3	0.7	1.1	2.9	1.0	1.0	2.0	0.0	0.1
Field size^b^	0.3	0.7	1.4	0.0	0.5	0.0	1.1	0.0	7.0
Field slope^c^	0.2	0.0	0.1	0.5	0.2	0.1	0.2	0.0	0.5
Soil type^d^	2.1	1.1	0.5	1.8	1.0	3.0	0.0	0.0	1.9
Soil type × Year	1.0	4.6	2.3	0.0	3.7	2.9	1.5	2.4	1.5
Pre-crop	4.9	7.4	13.4	5.2	5.2	0.0	0.1	0.0	0.0
Pre-crop × Year	10.8	2.9	3.9	4.3	13.7	10.2	17.7	11.7	19.2
Year	3.3	3.1	5.5	4.2	0.0	6.4	3.9	8.0	2.0

*^*a*^farm size categorized as 0–30, 30–60, 60–100, and >100 ha. ^*b*^field size as <0.5, 0.5–1.0, 1.0–3.0, 3.0–5.0, and >5 ha. ^*c*^field slope as <0.6, 0.6–1.3, 1.3–3.2, and >3.2%. ^*d*^soil type as coarse mineral soils like *Haplic Podzol 1* and *2*, clay soils like *Vertic Cambisol*, clay soils like *Eutric Cambisol, Gleyic Cambisol* and *Gleysols*, and peat soils.*

## Results

**FIGURE 3:**
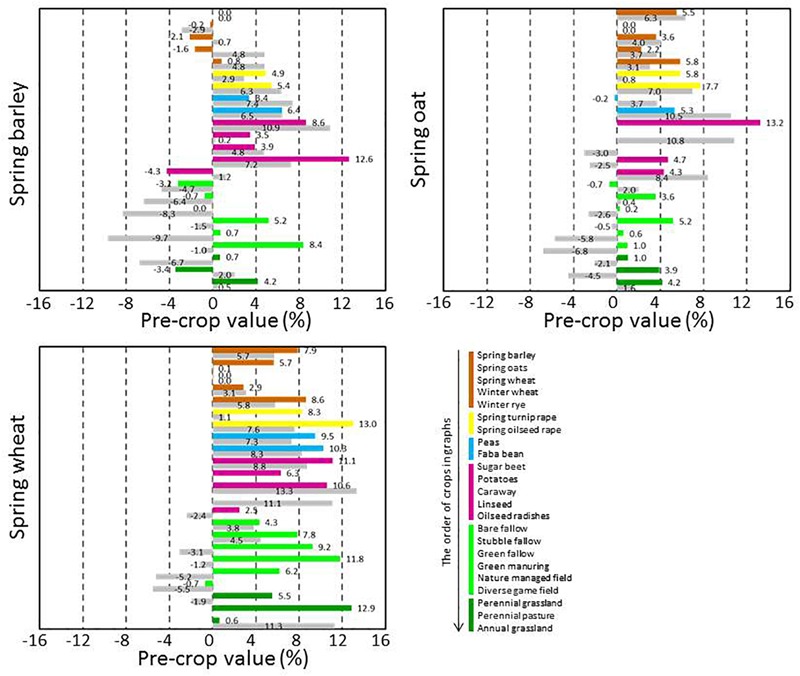
Pre-crop values (% compared to the case for the same pre- crop and following crop) for subsequent spring barley, oats, and wheat in 2016 (bar in color) and in 2017 (lower bar in gray). Results are shown only when the number of cases for each pre-crop and subsequent crop combination was ≥20. For barley the number of observations ranged from 21 to 6,890 depending on year and pre-crop, for oats from 25 to 5,925 and for wheat from 23 to 6,887. Barley preceded barley in 15,530 and 12,844 cases, oats preceded oats in 9,519 and 9,781 cases and wheat preceded wheat in 8,244 and 7,560 cases in 2016 and 2017, respectively.

**FIGURE 4:**
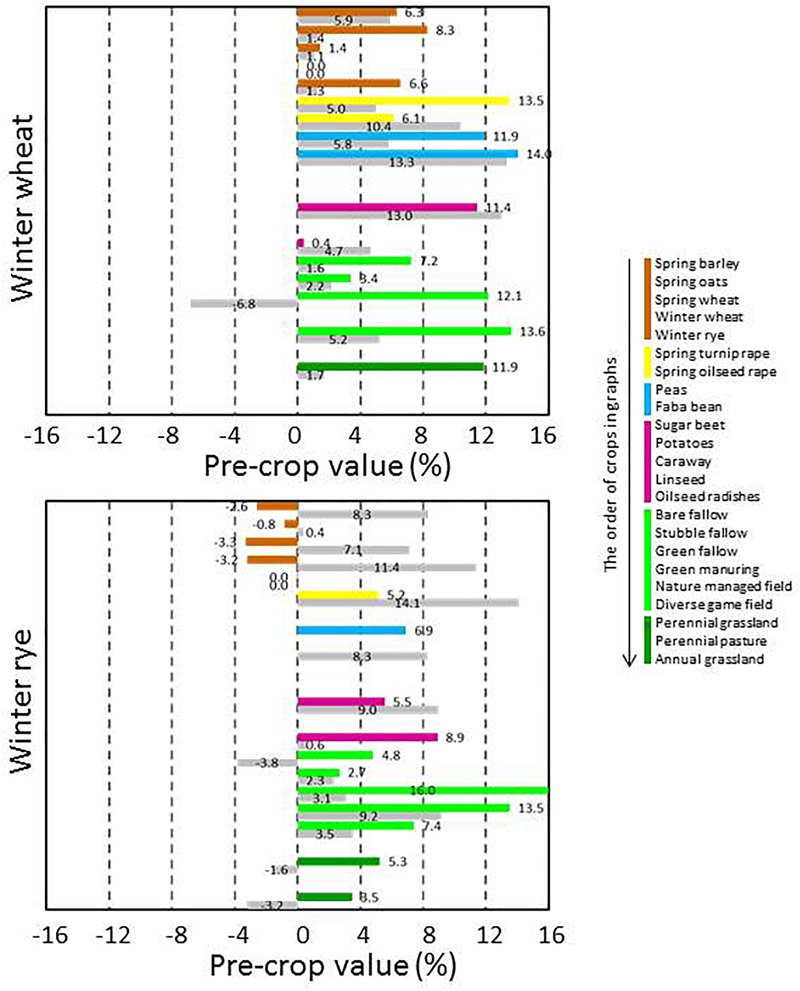
Pre-crop values (%) for subsequent winter wheat and rye in 2016 (bar in color) and in 2017 (lower bar in gray). For wheat the number of observations ranged from 20 to 2,039 depending on the pre-crop and year, and for rye from 20 to 1,089. Wheat preceded wheat in 1,044 and 619 cases and rye preceded rye in 754 and 452 cases in 2016 and 2017, respectively.

**FIGURE 5:**
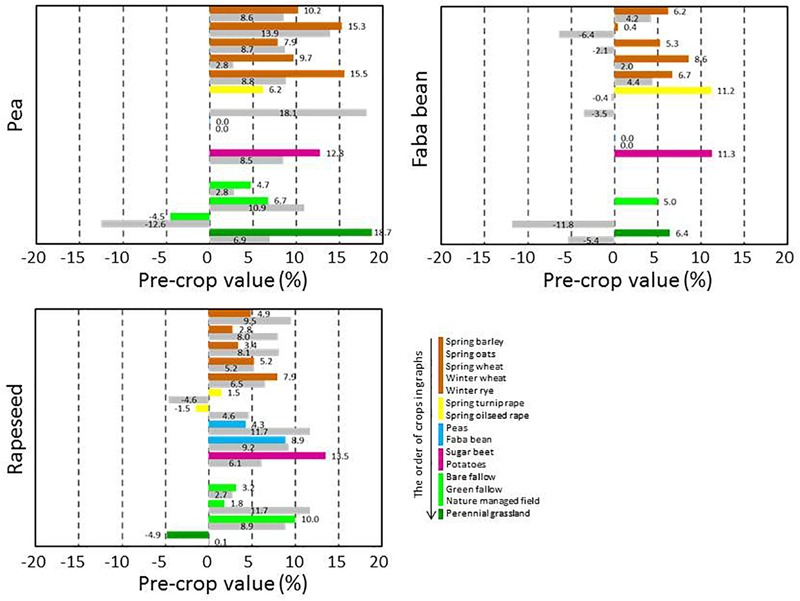
Pre-crop values (%) for subsequent spring rapeseed (both turnip rape and oilseed rape), peas and faba beans in 2016 (bar in color) and in 2017 (lower bar in gray). For rapeseed the number of observations ranged from 24 to 1,771 depending on year and pre-crop, for peas from 21 to 555 and for faba beans from 21 to 854. Rapeseed preceded rapeseed in 66 and 110 cases, peas preceded peas in 176 and 168 cases and faba beans preceded faba beans in 92 and 137 cases in 2016 and 2017, respectively.

**FIGURE 6:**
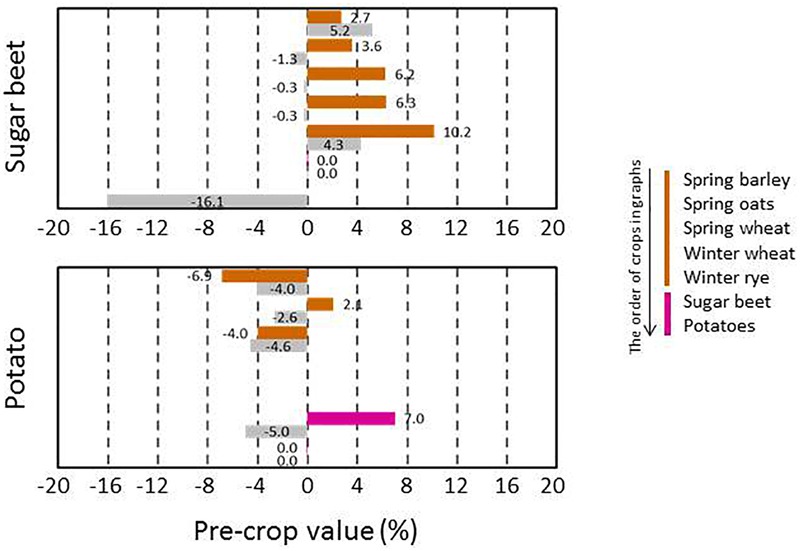
Pre-crop values (%) for subsequent sugar beet and potato crops in 2016 (bar in color) and in 2017 (lower bar in gray). For sugar beet the number of observations ranged from 29 to 211 depending on year and pre-crop and for potatoes it was from 31 to 64. Sugar beet preceded sugar beet in 413 and 383 cases and potatoes preceded potatoes in 328 and 301 cases in 2016 and 2017, respectively.

**FIGURE 7:**
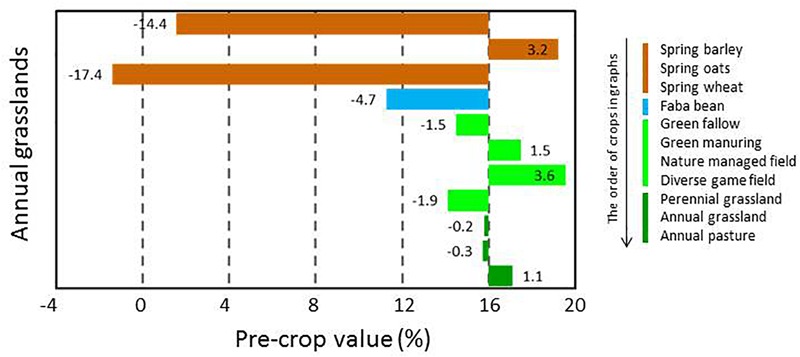
Pre-crop values (%) for subsequent annual production grassland in 2016 (data not available for 2017). The number of observations ranged from 20 to 360 depending on the pre-crop and the annual grasslands were established after annual grassland in 147 cases.

The pre-crop values were available for a high number of pre-crop and subsequent crop combinations, but the availability of pre-crop data varied depending on following crop. In general, pre-crop choices were more exhaustive for spring barley and wheat than for oats (Figure 3), winter wheat and rye (Figure 4), grain legumes and rapeseed (Figure 5), sugar beet and potato (Figure 6), and annual grasslands (Figure 7).

Rapeseed, grain legumes, sugar beet, potatoes and special crops such as caraway, linseed and oilseed radishes were very beneficial pre-crops for spring cereals (Figure 3). The pre-crop value of these previous crops varied depending on the year, in most cases this was ≤3% per unit for spring barley and wheat. However, it was the opposite for oats, for which the pre-crop values varied more. In contrast to oats and wheat, for barley the pre-crop values of other cereals (spring or winter types of cereals), were often negative and they tended to be lower than for non-cereal species. The pre-crop values of different types of fallow and production grassland were variable, and often even opposite depending on year: positive in 2016 but negative in 2017. For winter wheat, virtually all other pre-crops were more beneficial than the winter wheat itself (Figure 4). The pre-crop values of other cereals for winter rye again varied depending on the year. In 2016 the effect was negative, while in 2017 it was positive. Bare fallow was often less beneficial compared to other fallow types for subsequent spring cereals.

Cereals had positive pre-crop effects on peas and rapeseed and also quite frequently for faba beans (Figure 5). Rapeseed and sugar beet had positive impacts on the growth of subsequent grain legumes. Sugar beet and potatoes are grown a lot in monocultures and hence, the number of available pre-crops was limited. All cereals tended to have either positive or only negligible pre-crop values for sugar beet (6–10% at most), while they were mostly negative for potatoes (Figure 6).

Data on annual grasslands was available only for 2016 and with a limited number of pre-crops. It appeared that annual grasslands were often established right after annual grasslands without any break-crop. Spring barley and wheat were the least suitable previous crops (Figure 7), followed by faba beans. Different types of grasslands had only marginal impacts as pre-crops for annual grasslands.

**FIGURE 8:**
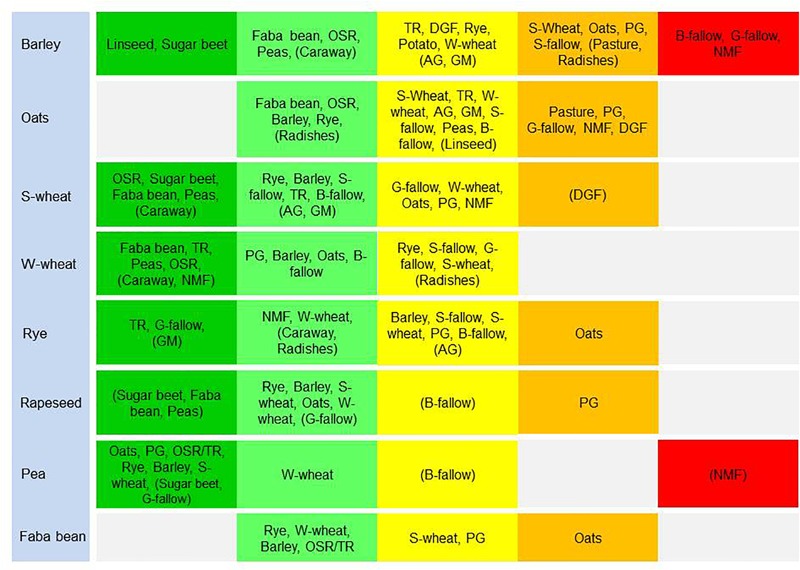
The rank orders of different crops and/or form of land use according to their pre-crop value for 2 years for spring and winter cereals, rapeseed, peas, and faba beans. The dark green indicates pre-crop values of ≥8.0, light green indicates ≥4.0, yellow indicates >0.0, orange indicates >–4.0 and red indicates ≤–4.0. Different pre-crops are shown in their rank order starting with the highest value. Pre-crops with data on <100 field parcels are shown in parenthesis. Pre-crop values are not shown if the value is available only for 1 year. OSR, spring oilseed rape; TR, spring turnip rape; *S*-wheat, spring wheat; *W*-wheat, winter wheat; *B*-, *S*- and *G*-fallow, bare, stubble and green fallow, respectively; GM, green manuring; NMF, nature managed field; DGF, diverse game field; PG, perennial grassland; and AG, annual grassland.

On the basis of the data available in Figure 3–7, previous crops were ranked according to their pre-crop value (Figure 8). Thereby, the generally most beneficial previous crops were identified. These often included grain legumes, rapeseed and sugar beet. By acknowledging the pre-crop values, several potential 5-year crop rotations were characterized (Table 2): when maximizing the pre-crop benefits, grain legumes and rapeseed were included in virtually all of the most advantageous rotations.

**Table 2:** Examples of potential 5-year crop rotations (each crop only once in a 5-year period) that make full use of the estimated pre-crop values and include the primary crop choices cultivated in the study region.

Examples of potential 5-year crop rotations	Pre-crop values (%)
**Grain crop rotation**:	
GL →*S*-wheat → Rapeseed →*W*-wheat → Rye ^∗^	A → B → A → B → A
GL → Rapeseed →*W*-wheat → Rye →*S*-wheat ^∗^	A → A → B → B → A
GL → Rapeseed → Barley →*W*-wheat → Rye	A → B → B → B → A
GL → Rapeseed → Oats →*W*-wheat → Rye	A → B → B → B → A
GL →*W*-wheat → Rye → Rapeseed →*S*-wheat^∗^	A → B → B → A → A
GL →*W*-wheat → Rye →*S*-wheat → Rapeseed^∗^	A → B → B → B → A
GL →*W*-wheat → Rye → Rapeseed → Barley/Oats^∗^	A → B → B → B → A
GL →*W*-wheat → Rapeseed → Rye →*S*-wheat ^∗^	A → B → A → B → A
GL →*W*-wheat → Rapeseed → Barley →*S*-wheat^∗^	A → B → B → B → A
GL → Barley →*S*-wheat → Rapeseed → Rye ^∗^	B → B → B → A → A
GL → Barley →*W*-wheat → Rapeseed →*S*-wheat/Rye ^∗^	B → B → B → A → A
GL → Barley → Rapeseed →*W*-wheat → Rye ^∗^	B → B → A → B → A
GL → Oats →*W*-wheat → Rapeseed →*S*-wheat/Rye ^∗^	B → B → B → A → A
GL → Oats → Rapeseed →*W*-wheat → Rye ^∗^	B → B → A → B → A
**Rotation with 2-year caraway**:	
Caraway I+II →*S*-wheat → GL → Rapeseed/*W*-wheat^∗^	A → A → A → N.A.
Caraway I+II →*S*-wheat → GL → Barley/Oats	A → A → B → N.A.
Caraway I+II →*S*-wheat/*W*-wheat → Rapeseed → GL/*W*-wheat/Rye^∗^	A → B → A → N.A.
Caraway I+II →*W*-wheat → Rye → GL	A → B → A → N.A.
Caraway I+II →*W*-wheat → GL →*S*-wheat/Rapeseed	A → B → A → N.A.
Caraway I+II → Barley → GL →*S*-wheat/*W*-wheat/Rapeseed^∗^	B → A → A → N.A.
Caraway I+II → Rye → GL → Rapeseed/*S*-wheat/*W*-wheat^∗^	B → A → A → N.A.
**Rotation with 2-year GF**:	
GF I+II → Rye → GL → Rapeseed/*S*-wheat/*W*-wheat^∗^	A → A → A → N.A.
GF I+II → Rye → GL → Barley/Oats	A → A → B → N.A.
GF I+II → Rye →*S*-wheat → GL	A → B → A → N.A.
GF I+II → Rye → Rapeseed → GL/*S*-wheat/*W*-wheat^∗^	A → B → A → N.A.
GF I+II → GL → Rapeseed →*S*-wheat/*W*-wheat/Rye^∗^	A → A → A → N.A.
GF I+II → GL →*S*-wheat/*W*-wheat → Rapeseed^∗^	A → A → B → N.A.
GF I+II → GL →*W*-wheat → Rye^∗^	A → A → B → N.A.
GF I+II → GL → Rapeseed → Barley/Oats	A → A → B → N.A.
GF I+II → Rapeseed →*S*-wheat/ Rye → GL ^∗^	B → A → A → N.A.

*Pre-crop values for each 5-year period are shown as sequential means across 2016 and 2017 (N.A. means not available for the specific previous and subsequent crop combination). GL, grain legume (faba bean and/or pea); GF, green-fallow; *S*-wheat, spring wheat; and *W*-wheat, winter wheat. Pre-crop values (%) are shown in the same order as crops in rotation and the last value is the pre-crop value of the last crop in rotation for the first crop: the letter A means pre-crop value is ≥8% and B 4–7.9%. Only rotations with clear positive impacts (and at least two times a pre-crop with value ≥8%) on the subsequent crop in rotation are shown. ^∗^Depending on region and growing season conditions, oilseed rape and faba bean may mature too late for autumn sowing of winter rye or wheat.*

Weather conditions differed during the study years (Supplementary Figure S1) and varied from warm and dry in 2016 to cool and rainy in 2017. Because the pre-crop values tended to vary, we estimated the main sources of variation in the NDVI-gap values, the source of the estimation of the mean pre-crop values. The coefficient of determination was moderate accounting up to half of all the variation: 36% for barley, 34.5% for oats, 37.4% for wheat, 40.5% for winter wheat, 46.3% for winter rye, 50.6% for rapeseed, 50.1% for faba beans, 47.3% for sugar beet and 41.8% for potatoes. The main identified source of the variation was the farmer, who alone contributed some 61–76% of the recorded variation (Table 1). This means that farms differed significantly in their NDVI-gap values because of different decisions made by farmers. Furthermore, pre-crop, year and pre-crop × year interaction caused variations in the NDVI-gaps especially for cereals. From the field parcel characteristics the soil type accounted for the variation in the NDVI-gaps depending on the crop: e.g., by 4.9 and 10.8% for spring barley, 13.4 and 3.9% for spring wheat and 5.2 and 13.7% for winter rye for the soil type and soil type × year, respectively. For other crops than cereals, the pre-crop was not a source of variation in the NDVI-gap values. This was in contrast to the pre-crop × year interaction, which accounted for some 10–19% of the variation.

## Discussion

Because of the knowledge gap on the pre-crop value of various pre-crop and subsequent crop combinations (Jensen et al., 2010), the motivation of this study was to develop a novel method to fill the gap. An NDVI-value based method may substitute to some extent the need for arranging large-scale multi-locational, year and factor experiments in the future, as such experiments are very resource intensive and expensive. Information on the value of previous crops for a high-number of following crops is urgently needed, though not only to complement the currently scarce and scattered data on pre-crop values (Preissel et al., 2015; Sieling and Christen, 2015), in which wheat is a frequent subsequent model crop (McEven et al., 1989; Persson et al., 2008; Angus et al., 2015; Schillinger and Paulitz, 2018) and only occasionally are other crops such as barley and rapeseed considered (Sieling and Christen, 2015). Only by providing data for various pre-crop and subsequent crop combinations, will large-scale planning of beneficial and also profitable crop sequencing patterns be possible for a farmer to support diversification of the current monocultural land use (Peltonen-Sainio et al., 2017).

### Pre-crop Values and Their Benchmarking With Those From Field Experiments

In general, pre-crop values based on the estimation of NDVI-gaps indicated that often any other crop than the subsequent crop itself had a positive pre-crop value. Rapeseed always had a positive pre-crop value for cereals and grain legumes, and so did peas and faba beans for cereals and rapeseed. The benefits were usually >5%, but in some cases even ≥10% (e.g., for spring or winter wheat after grain legumes or rapeseed). Depending on the year and the pre-crop and subsequent crop combination, not only differences in pre-crop values but also fluctuation between negative and positive were found, as was also apparent in field experiments (McEven et al., 1989; Kirkegaard et al., 1997). Ranking of the pre-crops according to their value for subsequent crops further highlighted the repeated benefits of grain legumes, rapeseed and sugar beet for cereals (Figure 8). However, some limitations to pre-crop choices may occur due to the short growing season of the high-latitude conditions: e.g., there are not necessarily many pre-crop choices for early sown winter rye as grain legumes and oilseed rape may be too late maturing (Table 2).

Many crop specific differences in pre-crop values occurred. For winter wheat, virtually anything other than winter wheat itself provided pre-crop benefits. The pre-crop benefit was the highest for faba beans, turnip rape, green fallow and nature managed fields, perennial grasslands and caraway with up to a 14% increase in the pre-crop value for the subsequent winter wheat (rough estimate ∼700 kg ha^-1^). The effects on the yield were less in the short growing season of the high latitudes than in experiments elsewhere (McEven et al., 1989; Sieling and Christen, 2015), though they were comparable to some other studies including Swedish experiments (Angus et al., 2015). For spring oats and wheat, any grain, seed, tuber or root crop had a positive pre-crop value in both years ranging typically from 3% to 8% and was higher for wheat (rough estimate ∼400 kg ha^-1^ at most). This agreed also with the results for barley, except that negative pre-crop values for barley were also found. Different types of grasslands and fallow fields had mainly positive effects on the following winter cereals. However, they tended to be the main group of crops with negative pre-crop values in addition to some cereals (Figure 8). Cereals had a positive pre-crop value for sugar beet especially in 2016, but for potatoes the pre-crop effects of cereals and sugar beet were variable without any clear indication of positive impacts. It is possible that the abundancy of above-ground biomass, characterized by the NDVI-values, does not associate well with tuber yields, which causes uncertainty in estimations of the pre-crop values. The harvest index (HI) for potatoes is high and quite variable (Chang et al., 2016, 2018) compared to that of cereals, rapeseed and grain legumes (Peltonen-Sainio et al., 2008; Gómez et al., 2017; Storer et al., 2018). The value of spring barley, wheat and faba beans as pre-crop for annual production grasslands was negative, but data was scarce and available only for one year.

### Limitations of the Developed Method for the Estimation of Pre-crop Values

The estimates are based on NDVI-values derived from Sentinel-2 as field parcel averages. Hence, the pre-crop values indicate the general growth capacity of each field parcel. This is not fully comparable to the harvested yield in the grain, seed, tuber and root crops in contrast to different types of grasslands in which cases the above-ground biomass is harvested. On the other hand, the transformation of the mean NDVI-value of a field parcel into the harvestable yield is likely to cause some uncertainty because of the growing conditions, management, the crop and cultivar dependent differences in partitioning (Peltonen-Sainio et al., 2008; Storer et al., 2018). For example, in Finland HI ranges by some 20 percent unit for spring cereals (being 40–60%) depending on soil type, growing conditions, cultivar and their interactions (Peltonen-Sainio et al., 2008). Hence, this developed method for the estimation of pre-crop values is based on the general growth capacity of a subsequent crop on a field parcel scale without considering partitioning between harvestable and non-harvestable parts of the above-ground biomass.

The developed method for estimating pre-crop values is dependent on availability of data for different pre-crop and following crop combinations. The pre-crop value of perennial grasslands to other crops can be estimated with this method but not vice versa, because of biased outcomes (Supplementary Figure S2) when the pre-crop value is estimated for the 1st year harvest, which typically produces a lower yield than the subsequent year harvest. Nonetheless, the pre-crop value of grasslands to other crops is more relevant than the pre-crop value of other crops for the grasslands. Furthermore, there is insufficient data on crops mostly cultivated in monoculture rotations for estimation of the value of a high number of alternative previous crops, even though the dataset used in this study covers in total ∼240.000 field parcels. This was especially striking for potatoes and sugar beet, for which spring and winter cereals were the only pre-crops with sufficient data available to estimate their value for a subsequent potato or sugar beet crop. The same was also true for faba bean as a following crop, which is mainly attributable to their role as a minor crop, however, with a gradually expanding cultivation area^4^. Pre-crop choices were also limited for annual grasslands. Usually other types of grasslands and spring cereals preceded them. Scarce knowledge on pre-crop choices may limit a farmer’s actions toward diversification of monocultural potato and sugar beet sequencing. These findings underline that the future experiments should focus on estimating pre-crop values for previous and following crop combinations which suffer from insufficient on-farm data.

Because the developed method is based on datasets on the field parcel scale, the evaluation of all the potential drivers causing variation in NDVI and pre-crop values is limited. Nonetheless, estimation of the impacts of weather conditions, soil type and numerous physical field parcel characteristic on the variation in pre-crop values is feasible, although the most challenging is for precipitation. However, the impacts of crop management practices are beyond reach of this method, though they are important (McEven et al., 1989). The application rates of fertilizers, tillage methods (conventional, reduced, and direct drilling) and the use of crop protection methods are examples of practices that could further increase our understanding of factors causing variation in pre-crop values. It is likely that in Table 1 such management practices are primarily embedded in the impacts of farmers as a source of variation in the NDVI-gaps (Supplementary Figures S3–S7).

### Advantages of the Method

This method was developed to close the knowledge gap on pre-crop values and thereby, to complement or even substitute the need for field experiments focusing on pre-crop values as much as possible. Traditionally, immense experimental arrangements in crop rotation studies are needed to enable implementation of their outcomes in time and space (St-Martin et al., 2017). Due to this and the high degree of resource intensiveness of such studies, there is a lack of data on pre-crop values for a high number of pre-crop and subsequent crop combinations. The developed method can be rapidly applied to different regions within a country as well as across countries and continents for the estimation of pre-crop values for relevant, region-dependent crop choices, provided that the data is available on crops on the field parcel scale for each year. With a higher number of years available for analyses (Sentinel-2 data available since 2016), this method can be applied to also assess the impacts of different cropping systems on yields (St-Martin et al., 2017). An evident additional advantage is that the data originating from actual farmer’s fields (i.e., conditions where the actual production takes place) is also more credible for the farmer, compared to experimental arrangements that often have more dedicated care and attention (Weiser et al., 2018). This is likely to support the implementation of this understanding of pre-crop values.

In addition to enabling the estimation of pre-crop values for a high number of pre-crop and subsequent crop combinations, which is the indisputable superior advantage of the developed method, another evident advantage is that the estimates of pre-crop value can be updated annually in a resource-efficient manner, benefitting from the automated system provided by the Finnish Geospatial Institute. So far we have used data on ca. 240,000 NDVI values on a field parcel scale and have limited this study to a region with the best opportunities for cultivating diverse crop choices. In the next steps we will expand the study to also cover more inland, eastern and northern regions of the country. This is likely to expand the data to cover millions of NDVI-values. In those regions, the number of different pre-crop and following crop combinations is, however, more limited due to the shorter growing season. By analyzing the data from forthcoming years we will be able to better identify the primary reasons causing variation in the NDVI-values, and gaps and thereby, pre-crop values, which again will provide additional understanding for the farmer. This in turn will help lead to condition-specific tailoring of land use and diversification. High number of consecutive years of NDVI-values for each field parcel enables estimation of pre-crop values depending on rotation type. This information supports planning of the most advantageous crop rotations, which were only shown as examples in Table 2 based on two year results.

## Conclusion

In conclusion, with the developed Sentinel-2 data-based method, for the first time we have produced estimates of pre-crop value for a high number of previous and subsequent crops combinations. The pre-crop values followed the general understanding and published results on the benefits of different crops as pre-crops. With this novel method, dynamic pre-crop values can be updated every year with new data, provided that the information on crops grown in each parcel is available as it is in Finland. When data on more years and also beyond our test region are available, further understanding of the pre-crop value dependency on growing conditions can be gained, which will further support the implementation of diversification by farmers. This study highlights the opportunities available when benefitting from digitalization. The developed novel method may enable the replacement of most of the currently used, highly resource intensive field experimentation with the on-farm data that originates from Sentinel-2 derived NDVI-values on the field parcel scale.

## Author Contributions

PP-S and LJ conceived and designed the study and interpreted the results. LJ designed the methodology and performed the statistical analyses. EH, SW, MK, and EP processed satellite data and provided NDVI-values. All the authors contributed to writing, PP-S as the leading author.

## Conflict of Interest Statement

The authors declare that the research was conducted in the absence of any commercial or financial relationships that could be construed as a potential conflict of interest.

## Footnotes

1 www.ruokavirasto.fi/en

2 http://step.esa.int/main/third-party-plugins-2/sen2cor/

3 http://step.esa.int/main/toolboxes/snap/

4 http://stat.luke.fi/en/
