# Supplementary material for: Pre-crop Values From Satellite Images for Various Previous and Subsequent Crop Combinations
Source: Front Plant Sci. 2019 Apr 9;10:462. doi: 10.3389/fpls.2019.00462 (PMC6465551; doi:10.3389/fpls.2019.00462)
Supplement: Supplementary file 1 [file Table_1.DOCX]

Supplementary Material

Pre-Crop Values from Satellite Images for Various Previous and Subsequent Crop Combinations

Pirjo Peltonen-Sainio^1*^, Lauri Jauhiainen^2^, Eija Honkavaara^3^, Samantha Wittke^3,4^, Mika Karjalainen^3^, Eetu Puttonen^3^

*** Correspondence:** Corresponding Author: pirjo.peltonen-sainio@luke.fi


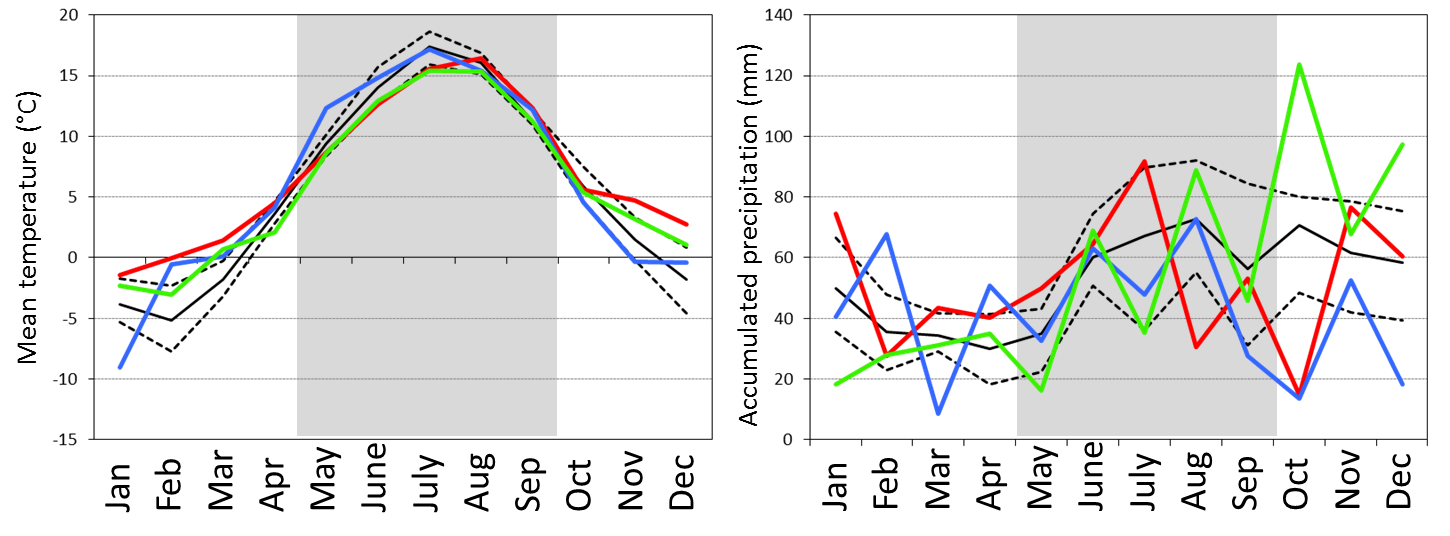


**Supplementary Figure 1.** Monthly mean temperatures (°C) and the accumulated precipitation for 2015 (red line), 2016 (blue line) and 2017 (green line). The black line indicates the mean for the 1991‒2014 period and the dashed black lines indicate the upper and lower quartiles. The grey area indicates the timing of the growing season.
