# Supplementary material for: Pre-crop Values From Satellite Images for Various Previous and Subsequent Crop Combinations
Source: Front Plant Sci. 2019 Apr 9;10:462. doi: 10.3389/fpls.2019.00462 (PMC6465551; doi:10.3389/fpls.2019.00462)
Supplement: Supplementary file 2 [file Table_2.DOCX]

Supplementary Material

Pre-Crop Values from Satellite Images for Various Previous and Subsequent Crop Combinations

Pirjo Peltonen-Sainio^1*^, Lauri Jauhiainen^2^, Eija Honkavaara^3^, Samantha Wittke^3,4^, Mika Karjalainen^3^, Eetu Puttonen^3^

*** Correspondence:** Corresponding Author: pirjo.peltonen-sainio@luke.fi


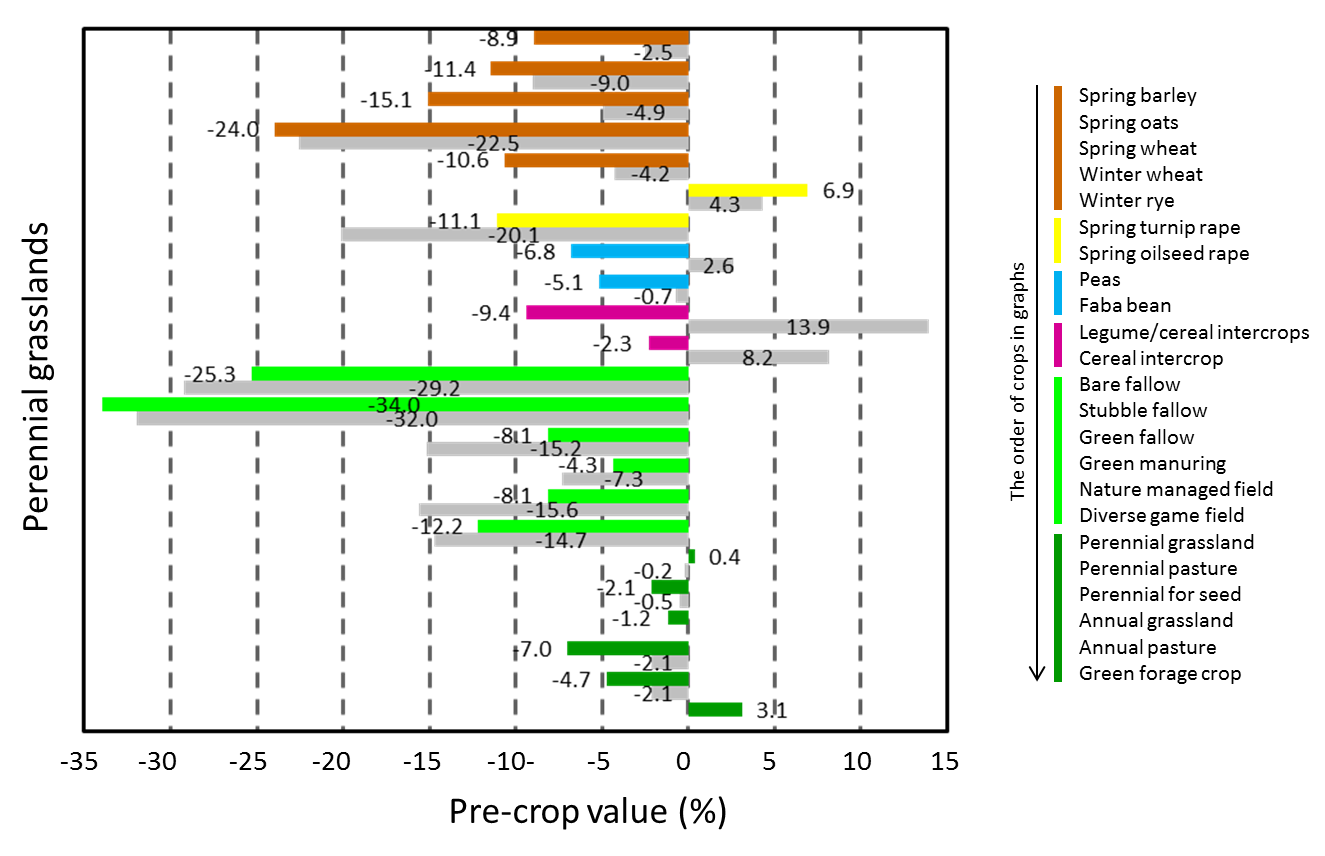


**Supplementary Figure 2.** Pre-crop values (%) for subsequent perennial production grassland in 2016 (bar in color) and in 2017 (lower bar in grey). The number of observations ranged from 20 to 2,282 depending on year and pre-crop. In16,786 and 18,320 cases in 2016 and 2017, respectively, production grasslands were followed by production grasslands, because grasslands are usually maintained for three to four years at a time.
