# Supplementary material for: Pre-crop Values From Satellite Images for Various Previous and Subsequent Crop Combinations
Source: Front Plant Sci. 2019 Apr 9;10:462. doi: 10.3389/fpls.2019.00462 (PMC6465551; doi:10.3389/fpls.2019.00462)
Supplement: Supplementary file 3 [file Table_3.DOCX]

Supplementary Material

Pre-Crop Values from Satellite Images for Various Previous and Subsequent Crop Combinations

Pirjo Peltonen-Sainio^1*^, Lauri Jauhiainen^2^, Eija Honkavaara^3^, Samantha Wittke^3,4^, Mika Karjalainen^3^, Eetu Puttonen^3^

*** Correspondence:** Corresponding Author: [pirjo.peltonen-sainio@luke.fi](mailto:pirjo.peltonen-sainio@luke.fi)


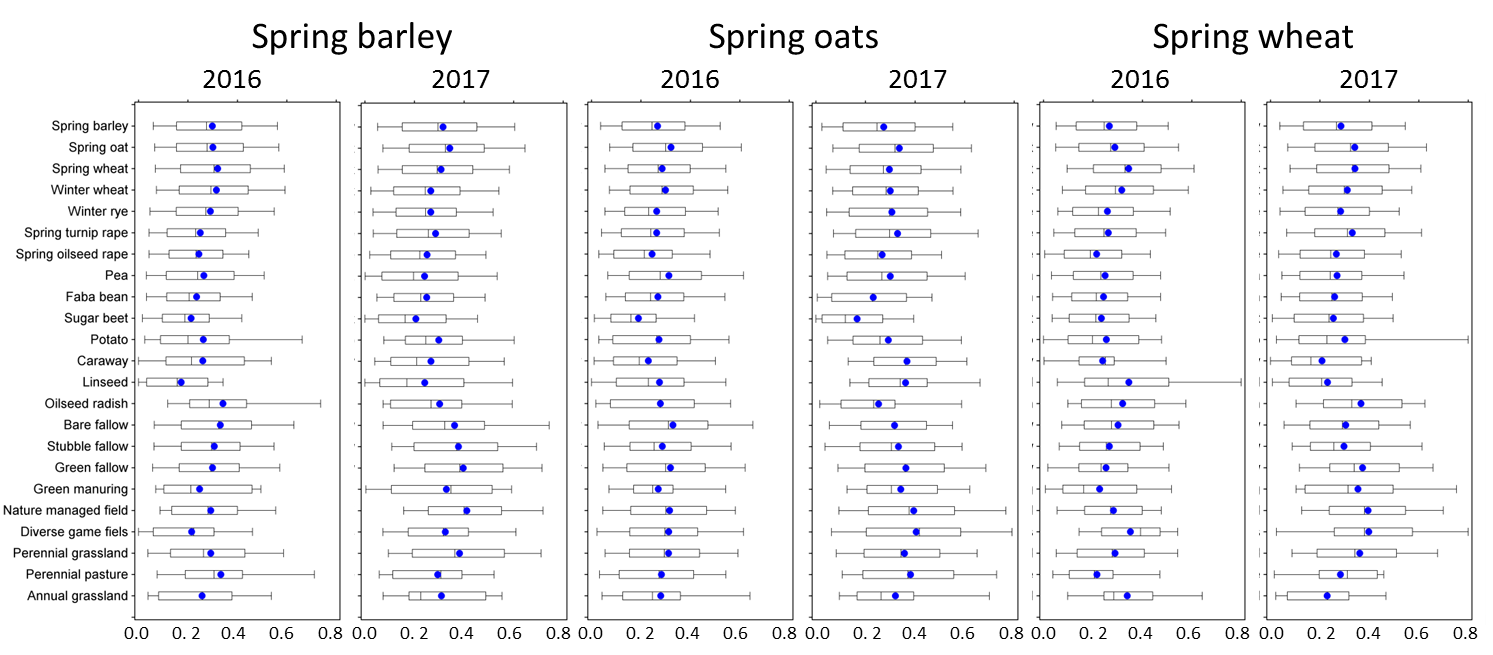


**Supplementary Figure 3.** Variation in the NDVI-gaps for different previous crops for spring cereals as subsequent crops. The boxes indicate the upper and lower quartiles, with the whisker tops at the 90^th^ percentile and whisker bottoms at the 10^th^ percentile. The solid lines inside the boxes are the medians and the blue dots indicate the mean value.
